# An IoT Surveillance System Based on a Decentralised Architecture

**DOI:** 10.3390/s19061469

**Published:** 2019-03-26

**Authors:** Amilcare Francesco Santamaria, Pierfrancesco Raimondo, Mauro Tropea, Floriano De Rango, Carmine Aiello

**Affiliations:** DIMES Department—University of Calabria, via Pietro Bucci cubo 39/c V piano, 87036 Rende (CS), Italy; p.raimondo@dimes.unical.it (P.R.); m.tropea@dimes.unical.it (M.T.); derango@dimes.unical.it (F.D.R.); aiello.carmine@outlook.it (C.A.)

**Keywords:** computer vision, surveillance, drones, internet of things, lightweight protocols, mist computing, OpenCV

## Abstract

In the last few years, we witnessed numerous episodes of terrorist attacks and menaces in public crowded places. The necessity of better surveillance in these places pushed the development of new automated solutions to spot and notify possible menaces as fast as possible. In this work, we propose a novel approach to create a decentralized architecture to manage patrolling drones and cameras exploiting lightweight protocols used in the internet of things (IoT) domain. Through the adoption of the mist computing paradigm it is possible to give to all the object of the smart ecosystem a cognitive intelligence to speed up the recognition and analysis tasks. Distributing the intelligence among all the objects of the surveillance ecosystem allows a faster recognition and reaction to possible warning situations. The recognition of unusual objects in certain areas, e.g., airports, train stations and bus stations, has been made using computer vision algorithms. The adoption of the IoT protocols in a hierarchical architecture provides high scalability allowing an easy and painless join of other smart objects. Also a study on the soft real-time feasibility has been conducted and is herein presented.

## 1. Introduction

In the last decade we saw a great effort of the research community in the surveillance and safety fields. These needs pushed the study and development of new solutions to faster detect and react to warning situations and menaces. The surveillance in public places like airports, bus stations and train stations have been greatly increased specially inside countries with high risks of terrorism. The technology improvement in embedded devices allowed the development of more powerful computing devices with small sizes that can afford to run basic analysis and recognition tasks on their own without relying on a cloud architecture. On the other hand, internet of things (IoT) protocol improvements led to the possibility to flawlessly manage networks with a huge number of nodes with small delay answer time and high stability. The conjunction of these two paradigms gave birth to an intermediate layer between the cloud and a local approach called “fog”. The fog approach is to not overload the cloud layer with all the tasks. The computational capability of the objects that compose the ecosystem takes care of the simpler tasks while the communication with the cloud is only used to run complex tasks or to send data needed for a further analysis. Giving a computational capability to the edge nodes allow a faster response to the situation where a cloud analysis is not needed. Even if the cloud analysis is necessary, it speeds up the process by starting the basic computation on the edge nodes saving in this way time and resources. In this work, we proposed to move some of the elaboration tasks on smart devices such as smart cameras for pre-elaborate collected data. In this way, it is possible to reduce elaboration time and reduce computational load on edge/fog nodes. This is possible because of the higher computational resources we can find on embedded devices. Thus, we decided to exploit these resources to initialize a computer vision (CV) [1,2,3] module on smart cameras for detection purposes. It is possible to find the same principle in the mist computing where some of the computing modules are moved on decentralized devices to reduce latency due to distance between end devices and computational modules [4]. Moreover, decentralising some of the functions system results in more scalable systems. The edge layer is responsible for collecting results of the decentralized node to classify events. It is also responsible for raising alarms and interacting with the human layer. In this work we proposed a system based on a decentralized architecture for increasing the scalability, the reliability and overall performances in terms of system response time. This decentralized architecture can be well developed for other applications such as UAV/drone networks and 5G/IoT user equipment (UE) designs. In [5] the authors provide an overview of UAV-aided wireless communications, by introducing the basic networking architecture and main channel characteristics, highlighting the key design considerations as well as the new opportunities to be exploited. In [6] the authors propose a hierarchical architecture of UAVs with multi-layer and distributed features to facilitate a smooth integration of different mainstream UAVs into the next-generation wireless communication networks. In [7] the authors first conduct an in-depth investigation of current technological trends of 5G from user equipment (UE), design perspective, and then present a costeffective cellular-WiFi design methodology based on the new distributed phased array MIMO (DPA-MIMO) architecture for practical 5G UE devices as an example. In [8] the authors present a hybrid UAV-WSN networks which are self-configured to improve the acquisition of environmental data across large areas. Their main contribution is the trajectory design, optimized to avoid interdicted regions, to pass near predefined way-points, with guaranteed communication time, and to minimize total path length. Other interesting works are [9] in which the authors try to fill the lack of facilities for including security policies by using a novel N-layered hierarchical context-aware aspect-oriented Petri net model that not only evaluates the drone behavior, but also assesses it for potential vulnerabilities by the utilization of security policies. In [10] the authors introduce a surveillance model for multi domain IoT environment, which is supported by reinforced barriers with collision-avoidance using heterogeneous smart UAVs. They propose a novel approach in order to solve the defined problem. The main contribution of this work are listed below:

- We proposed a decentralized architecture for reducing system response time and for increasing system scalability;
- We proposed a distributed protocol based on a well known machine-to-machine (M2M) protocol called message queuing telemetry transport (MQTT) [11];
- We defined three layers and for each layer we defined the role of each element;
- We designed and implemented each component of the system such as unmanned ground vehicle (UGV) and smart cameras;
- We implemented the application for interfacing human to the system by defining the human-to-machine (H2M) interfaces;
- We realized a testbed based on a real scenario.

The work is organised in the following way. In Section 2 the related works are discussed, here we raise up the main differences between our proposal and the others already published in the literature; In Section 3 the system architecture is presented. All elements of the architecture have been presented with a brief description of their main duties; In Section 4 we presented the protocol we used for allowing communication among layers and architectural elements. The protocol is based on a well known M2M protocol called MQTT which is based on the publisher/subscriber mechanism. In Section 5 we reported the results we obtained by evaluating the proposed solution in a well specified context. We presented the test-bed and we added a comparison of the proposal versus a edge/cloud based system. In Section 6 the conclusions of the work are presented. Here we point out the results we achieved, and a brief discussion about future works is provided.

## 2. Related Works

In the last years the researchers focused their studies on IoT environment with its protocols and algorithms in order to permit communication between different devices. Many works exist also in machine-to-machine, human-to-human and human-to-machine topics in order to explain how to allow communication in this domain showing the possible services and applications of the future scenarios.

### 2.1. IoT Domain

A lot of works exist on this topic. In [12] the new technology paradigm of IoT has been surveyed. The IoT is recognized as one of the most important areas of future technology and is gaining vast attention from a wide range of industries. The article presents IoT technologies that are essential in the deployment of successful IoT-based products and services and discusses IoT categories for enterprise applications used to enhance customer value. In [13,14] the authors present a fair survey of technologies, applications and future direction for IoT. Attention is given to the security aspects investigating the security capabilities of existing protocols and networking stacks for IoT. In [15] the authors provide an overview of IoT technologies required from an embedded design perspective and specific properties associated with IoT in embedded systems’ landscape. They investigate essential technologies for the development of IoT systems, existing trends, and its distinguishing properties. In [13] the authors show the narrow band technologies applied in the IoT domain. In particular, on the basis of 3GPP introduction of a new narrowband radio technology called narrowband IoT (NB-IoT) in release 13, the authors highlight some of the key features introduced in NB-IoT and presents performance results from real-life experiments. NB-IoT was designed to support very low power consumption and low-cost devices in extreme coverage conditions. NB-IoT operates in very small bandwidth and will provide connectivity to a large number of low-data-rate devices. The experiments carried out show the performance measurements using the SDR implementation of NB-IoT in a C-RAN environment. The authors show how a cloud radio access network is a good candidate for NB-IoT implementation. In [16] the authors present Whisper, an enabler for software defined networking (SDN) in low power and lossy networks. The centralized whisper controller of a network remotely controls nodes’ forwarding and cell allocation. This mechanism ensures the best possible in-band connectivity between the controller and all network nodes. In [17] the authors propose a novel MQTT with a multicast mechanism to minimize data transfer delay and network usage for the massive IoT communications. The proposed MQTT reduces data transfer delays by establishing bidirectional SDN multicast trees between the publishers and the subscribers by means of bypassing the centralized broker.

### 2.2. H2H, M2M, H2M Domain

In [18] the authors discuss the IoT paradigm for interconnected objects that are identifiable and equipped with sensing, computing, and communication capabilities, taking benefit of the long term evolution (LTE)/ long term evolution-advanced (LTE-A), cellular networks to support machine-type communication (MTC) and human-type communication (HTC). The article [19] presents several human factor theories relative to human–machine interactions and how they can explain driver behaviour using lane departure warning system (LDWS). The predictions made by these theories are confronted with empirical data collected on drivers interacting with LDWS. In paper [20], the authors present two approaches to mitigate the impact of M2M communication in LTE-A. They model such overloaded scenarios as a bankruptcy problem and apply two strategies to define how resources should be allocated. The simulation results show that our approaches present improvements in terms of energy efficiency, impact control of M2M over Human-to-human (H2H) and define priority among different classes of device. Ref. [21] discusses about user-centered design (UCD), an approach for creating human-machine interfaces that are usable and support the human operator’s tasks. Formal methods are tools that enable analysts to consider all of the possible system interactions using a combination of formal modeling, specification, and proof-based verification. This work describes a method that supports UCD by automatically generating formal designs of human-machine interface behavior from task-analytic models. In [22] the authors present a scheme to overlay M2M traffic over human-to-human (H2H) communication under an orthogonal frequency-division multiple access (OFDMA) framework. In [23] the authors propose a novel data traffic aggregation model and algorithm along with a new 5G network slicing based on classification and measuring the data traffic to satisfy quality of service (QoS) for smart systems in a smart city environment. In their proposal, 5G radio resources are efficiently utilized as the smallest unit of a physical resource block in a relay node by aggregating the data traffic of several M2M devices as separate slices based on QoS for each application.

### 2.3. Mist Computation Domain

Fog and Mist computing exploits computational resources of IoT devices located at the edge of the network [24,25,26,27,28,29]. This new infrastructure brings benefits related to latency and Internet bandwidth utilization when compared with Cloud computing. However, this scenario is quite challenging mainly due to the considerable heterogeneity of the devices and to the dynamicity of the network topology that adds new issues related to security, privacy, data availability, and service availability, among others. In [30] the authors focus on the problem of data availability and dissemination in this environment by proposing a distributed algorithm using ideas from evolutionary computation and epidemic models. In [4] the authors, considering that self-awareness, situation awareness, and attention are key enablers of efficient fog and mist computing, explore two central aspects of self-awareness-situation awareness and attention and how they facilitate the assessment of human physiological data in a prototype self-aware health-monitoring cyber-physical system (CPS) infrastructure. In [31] the authors present optimization techniques for Linux to provide isolation and high performance for the control plane of SDN. The new techniques are (1) separate execution environment, and (2) separate packet processing. They evaluate the proposed techniques and show that the maximum performance of a control plane increases by four times compared to the native Linux while providing strong isolation. In [32] the authors propose qCon, which is a QoS-aware network resource management framework for containers to limit the rate of outbound traffic in fog computing. They show qCon’s effectiveness in a real fog computing environment implementing qCon in a Docker container infrastructure on a performance-limited fog device—a Raspberry Pi 3 Model B board.

Many works in literature already faced the problems of suspicious left luggage inside crowded area. In [33] the authors proposed a detection and recognition multi-camera algorithm capable of alerting human operators when a suspicious object is left in the field of view of some cameras. Authors of [34] proposed a framework for anomaly detection in crowd scenarios considering both spatial and temporal anomalies and taking into account model appearance and dynamics of crowd patterns. In [35] the authors describe how an aerial surveillance system can be built using an unmanned aerial vehicle or a drone. It can be used in peace keeping activities and also real time monitoring of a place at any time of the day. The aim is to provide fast and efficient surveillance at an affordable rate so that it can be used widely at private, institutional and governmental level. Liao et al. in [36] propose a localized approach to abandoned luggage detection based on an efficient foreground-mask sampling technique. With this technique is possible to identify an abandoned luggage in a very fast way locating and tracking then the owner of the luggage.

## 3. Proposed Architecture

**Figure 1:**
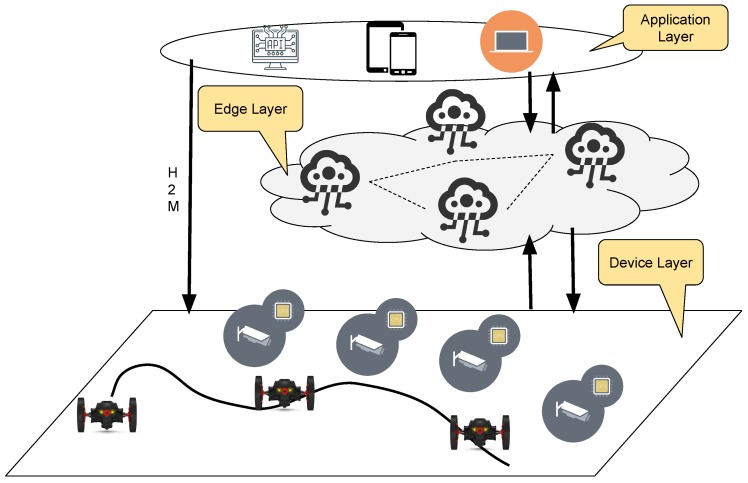
Decentralized reference architecture composed of a device layer, an edge layer and an application layer in which several instances can be executed for interfacing the system. Each application instance can receive the status of the system.

The goal of our work is to create a scalable system for monitoring efficiently indoor shared areas. Indeed, interests in surveillance framework raise in these last few years. Even more technologies can be applied in these contexts, and even more, devices can be used to accomplish specific tasks. Therefore, the management layer needs to be improved to fulfil requests coming from application layers. In this work, we designed a decentralized system which is based on edge and mist computing to realize a scalable architecture. The main core of the system is composed of an edge layer, it is responsible of elaborating data coming from sensors or decentralized smart objects such as camera, audio devices and drones. Moreover, the edge layer has to control mobile units by sending them commands or tasks to accomplish. The application layers, instead, can cooperate between them and they interact with the core layer for acquiring data about the device they want to queries or commands. Finally, the device layer is realized by using fixed or mobile devices. Devices which implements a computational module are considered to be smart, other devices used only for sensing or actuating purposes are instead considered not smart. In this work, we focus our attention on the design of a cognitive system able to understand the environment by itself by exploiting the devices ecosystem we provide. The Figure 1 depicts the reference architecture used in this work.

For better defining layer interactions we build up a mapping between the physical layer and logical layers. The lowest layer is called the environmental layer, and it has a direct connection with the physical layer composed of devices placed in the environment. The mid-layer is called the hidden layer, and it is composed of the computational unit we have in the system. The highest layer is called human layer, and it is composed of those applications that directly interface with the human. The logical layers we used are herein reported:

- Environmental layer;
- Hidden layer;
- Human layer.

**Figure 2:**
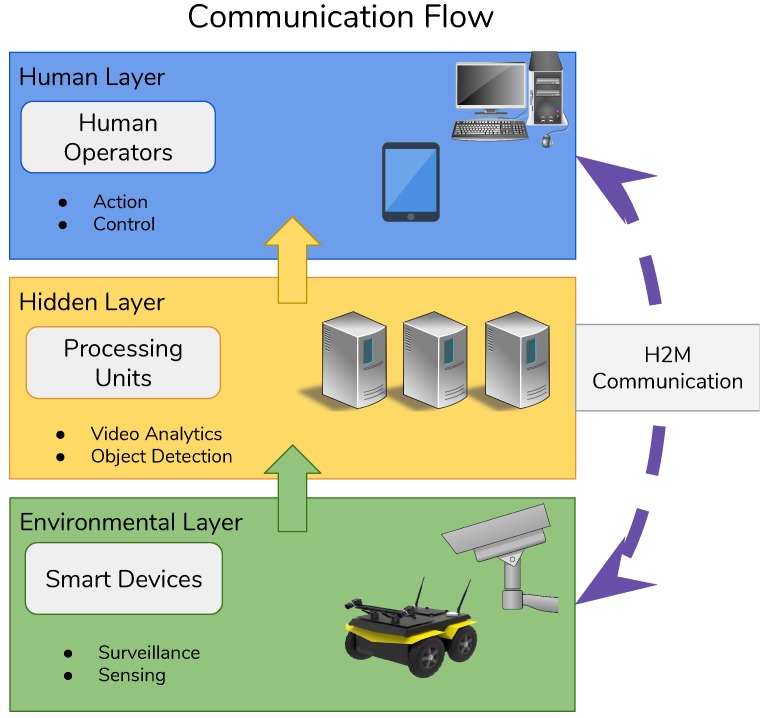
Three layers architecture: The orientation of communication flow is shown in this picture. Environmental layer communicates in a direct manner with the hidden layer and the human layer receives data from hidden layer. The data-flow travels from environmental layer to the human layer. The human-to-machine (H2M) interfaces are exploited to allow a direct connection between the humans and devices lying in the environmental layer.

Layers are organized as shown in the Figure 2. The environmental layer is the lowest one and it sends data to the hidden layer where the edge layer lies. Once data are collected and elaborated, the hidden layer brings up these data to the human layer. Humans can enter in the loop by using mobile devices or desktop for interacting with devices that lie in the environmental layer. Humans can either use direct interfaces by exploiting the H2M connections or he can exploit the hidden layer by modifying parameters to change system behaviour.

### 3.1. The Environmental Layer

The environmental layer is composed of several devices. Their task is to constantly collect data about the environment. These objects can be fixed cameras, UGV patrolling the area or even smart devices with several equipped sensors. Each of these objects have computational capabilities and can perform several task on their own.

### 3.2. The Human Layer

The human layer is composed by the human operators that inside the operative stations check the information obtained by the environmental layer. In the reference architecture, we showed in the Figure 1 the human layer matches with the application layer. Indeed, human interfaces are implemented in the applications that can interface the edge layer and the device layer. It is important to consider that in the current implementation and for security purpose only one user per time with administrative rights can directly interface the device layer.

### 3.3. The Hidden Layer

**Figure 3:**
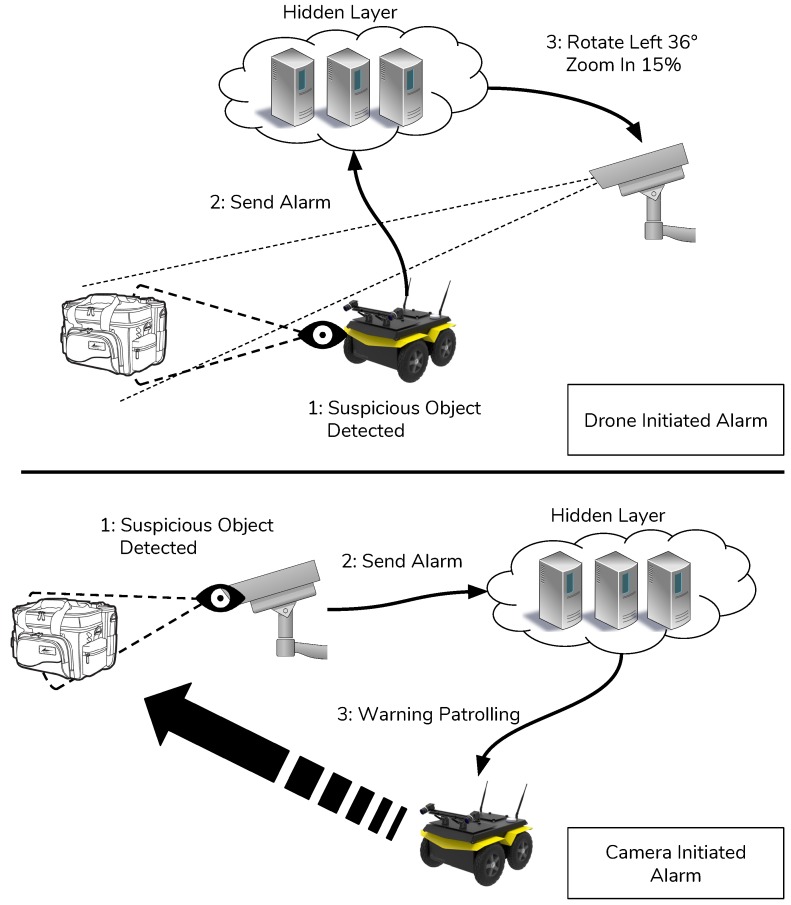
Hidden layer coordination function.

The hidden layer, see Figure 3, is composed by a cloud architecture that is always waiting for complex tasks assigned by the environmental layer. Every time an anomaly or a possible dangerous situation is identified it is reported to the human layer in order to validate it and eventually take the adequate countermeasures. The next subsections will show the configurations and the structure of the whole architecture.

### 3.4. Architecture Elements

In this section, we presented the different elements that compose the architecture applied in this paper. In particular, we studied the behaviour of the proposed system in a specific application to better evaluate its performances. Following the basic modules mentioned before, we used the following configuration. The lowest layer is composed of the following devices:

- Smart IP cameras: those devices can elaborate captured data, and if requested they can send to the higher layer pictures or video streaming. Commonly, IP cameras only sent results of the elaboration such as detected objects, location and alarms;
- unmanned ground vehicles (UGVs) devices: they are eligible for doing path-rolling in the environment and for doing focused researches on some specific location. They move in the environment autonomously by following a specific path. In case of needs, they can switch on free movements or pass under the control of a human by using H2M interfaces;
- sensor devices: they are installed in the area to be monitored, and they supply specific information such as presences or temperature, light intensity and so on;

The middle layer, also called the hidden layer, is composed of the edge/fog nodes that allow the system to interact with the physical lower layer also called device layer by exploiting direct interfaces or using MQTT protocol [37,38] messages we are going to specify in Section 4. The edge/fog nodes are connected each other by exploiting Internet connection. Moreover, this layer can be used by the application layer which queries for information and already elaborated data. Furthermore, the application layer supplies human interfaces. In the remainder of the section, we described each element of the architecture.

#### 3.4.1. Edge Computing

In this section we discuss of the design of the edge layer and which functions it implements. In particular, the edge layer is used to collect and elaborate data coming from the physical layer for helping the decision making algorithm to classify alarms to raise and to communicate to others. This physical layer gather all the data from the sensors dispatched inside the environment analysing them. The proximity of this set of computational nodes allow a faster response without slowing down the responsiveness of the system. The tasks of the edge computing layer are to integrate and further compute the data and information from different kinds of sensor. Using the information gathered from different device it is possible to give a better interpretation of the contexts and ensure a seamless coordination between the environmental layer devices. For example, if a sensor detects a presence inside a restricted area the system reacts instantaneously by sending one or more drone to check the situation. Furthermore, if an anomaly is detected by different devices in a certain area the attention of the system is raised in order to verify it as soon as possible. Edge layer uses resources for executing some tasks locally with the main goal of reducing latency in the elaboration nodes. Often latency is due because core nodes are placed in the cloud layer and they are far from the user instances of the application that require remote elaboration. Some previous studies showed that latencies may be reduced by migrating some delay sensitive computation module as closely as possible to the users [39,40]. The computation at edge cloud level is similar in functionality but different in terms of resources availability and elaboration delays. Indeed, the cloud layer uses a big pool of shared resources that bring up a higher computational capability to this layer, instead, edge layer has a limited set of resources but it is placed as closely as possible to the user layer. In this work, we evaluated the latency of the system in two different configurations, the first one presents only the cloud layer, the second one presents the edge layer as well. This configuration can be viewed as a M/M/c service model as shown in [41,42]. Here, each edge or core processes multiple service requests which are stored temporarily in a buffer with an assumed infinity capacity. Different policies may be used in the management of the buffer taking into account services requests priority. In that case, since we assume that requests may have certain latency constraints we can use a priority scheduling or if we treat all requests in the same way then we can use the first come first served (FCFS) scheduling. Finally, we can show that in the model we use, the latency depends of the arrival rate we called λ, the service rate we called μ and the number of available servers we called *c*. When the system load increases the arrival rate λ increases as well and we can measure a direct effect on the experimented latency. In particular, the computing latency per task which is called *V*. It is easy to demonstrate because of the following equation:

(1) $$d_{comp}=\frac{1}{\left(c\mu -\lambda \right)}$$

Here, the $d_{comp}$ term means the computation delay measured in a computation node that has an arrival rate of λ and a service rate of $\mu =\frac{f}{K}$. The *f* term means the instruction per seconds done by the computation node and the *K* term means the number of instruction required per task. Commonly the time spent of a generic task for performing its elaboration passing through a computation node is given by the sum of the transmission delay queue, delay and processing delay. Therefore, we can achieve the following equation:

(2) $$d_{node}=W+\frac{1}{\mu }+t_{tx}$$

Taking into account the service model ( M/M/c ) the *W* term which means queuing delay can be achieved by the next equation:

(3) $$W=\frac{{\left(c\rho \right)}^{c}}{c!}\times \frac{1}{1-\rho }p_{0}\times \frac{\rho }{\lambda \left(1-\rho \right)}$$

In the Equation (3) is shown the queue waiting time in a generic server node which is identified by an utilization factor called ρ and a queue probability called $P_{Q}$, in the Equation (4) we shown how it is possible to evaluate the $P_{Q}$. The initial queue probability is depicted in Equation (5).

(4) $$P_{Q}=\frac{{\left(c\rho \right)}^{c}}{c!}\times \frac{1}{1-\rho }p_{0}$$

(5) $$p_{0}={\left[\sum_{k=0}^{c}\frac{{\left(c\rho \right)}^{k}}{k!}+\frac{{\left(c\rho \right)}^{c}}{c!}\times \frac{1}{1-\rho }\right]}^{-1}$$

Finally, we can achieve the latency of the systems by assuming that the overall latency is composed of several terms such as propagation delay, transmission delay, routing node delays and processing delays on the computational nodes. For a cloud only model we can reuse the equation already proposed in [41]

(6) $$L_{cloud}=\alpha \times min_{i=0}^{m}\left(d_{ue,AP_{i}}\right)+\beta \times d_{AP,cloud}+d_{node}$$

At the same manner we can achieve the Edge based system latency:

(7) $$L_{edge}=\alpha \times min_{i=0}^{m}\left(d_{ue,AP_{i}}\right)+d_{node}+d_{s}$$

To better understand the next step we have to specify that with the term smart device we mean both UGV and smart camera. Thus, let us to define the α term, it is a proportional constant for the uplink and for the downlink bandwidth from the smart device to the access point (AP). At the same way the β term is the proportional constant for the uplink and for the downlink bandwidth from the AP to the cloud. The $d_{ue,AP_{i}}$ is the distance from the *i*-th AP to the smart device. The $d_{AP_{i},cloud}$ term is the distance between the *i*-th AP to the cloud. Moreover, the *m* term is the number of the available AP. Regarding the Equation (7), the term $d_{s}$ is the delay that a task can found due to an overload on the chosen edge node. We assume that this terms is equal to 5 ms as suggested in [41]. The list of the symbols used in the mathematical formulation are shown in Table 1.

#### 3.4.2. Unmanned Ground Vehicle (UGV)

**Figure 4:**
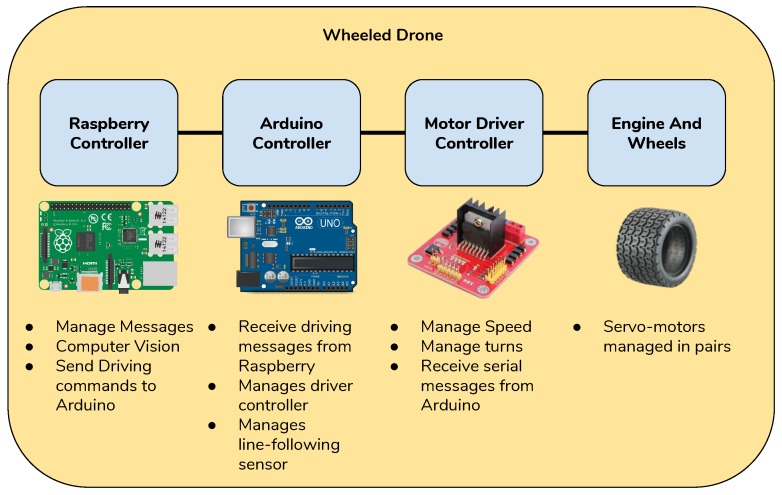
Unmanned ground vehicle (UGV) boards components and logic connections.

**Figure 5:**
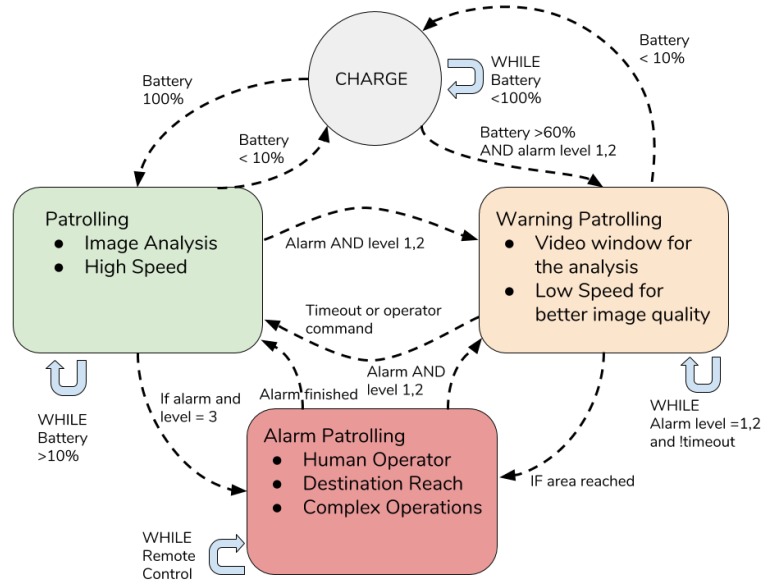
Finite state machine (FSM) of the drone.

In this section, we will show the configuration of each drone that is used to patrol areas inside the architecture. Each drone has been assembled using common components available on the market. A schematic representation is shown in Figure 4. In the following, we will describe how the components, depicted in the figure, work. The controller used for wheeled ground drone was Arduino Uno [43] and the mobility was ensured by four 9V electric engines connected to the control board *Motor Driver L298N*. The L298 was an all in one integrated circuit. It was designed to accept standard transistor–transistor logic (TTL) levels and drive inductive loads such as relays, solenoids, DC and stepping motors [44]. This kind of board allowed the separated management of only two engines and for this reason they have been connected in series. In this way it was possible to manage simultaneously two engines connected with the wheels on a side of the drone. The control board also manages a line following sensor bar that is needed to follow the circuit for the patrolling drone. The drone behaviour can be described by a simple finite state machine (FSM) with four states: charging, patrolling, warning patrolling, alarm patrolling. The default state of the drone was the patrolling state during which the drone simply follows the path-marks to move inside the patrolling area. During this state the drone was always searching for anomalies or suspicious objects. For a computer vision task, an Arduino Board did not possess the computational capabilities to run computer vision tasks. For this reason a Raspberry Pi 3 [45] was also equipped on board to run all the heavy tasks. When an anomaly is detected by the on-board algorithm the state changes in Warning Patrolling. During the warning patrolling state the drone slowed down to gather a more stable video stream and to allow a human operator to evaluate the situation. If no anomaly was detected or the human operator manually switched the state returns to patrolling. If the human operator detects a possible dangerous situation the state was changed to alarm patrolling. During the alarm patrolling, the drone was fully driven by a human operator to allow some very complex operations. From the alarm patrolling state the drone can switch to the warning patrolling or simple patrolling. The transition to the alarm patrolling state can also be triggered from a message from another smart object to notify the drone the area of interest to reach. The FSM schema is depicted in Figure 5.

#### 3.4.3. Smart Camera Devices

In this section, we discuss the IP camera devices that instantiated CV modules for detection purposes. It is composed of a custom IP camera plus an elaboration unit able to instantiate video analytic modules. In this work, we used a Sony fixed IP camera model *SNC-CH110*. This camera was able to capture data stream at the resolution of 1280 × 1024 pixels, it is good for day and night video capture. Moreover, this camera can be interfaced with a wired interface by exploiting on-board Ethernet port. The CV algorithm implemented and loaded on cameras has the task to detect and recognize objects by shape and colour. Each frame captured by the cameras undergoes several processing steps before the detection of possible objects to ease the recognition. These steps are:

- Space colour conversion;
- Colour and threshold definition;
- Morphological transformation;
- Outline detection.

The space colour of the frame was changed from RGB to HSV because the HSV colour space simplifies the detection and extraction of an object from a scene. The HSV space describes colours in terms of hue, saturation and intensity. The threshold step was a segmentation task to cluster the image on the basis of pixel intensity. This method output was a binary image where white pixels were considered interesting against a black background. The main operation was performed by defining a threshold value to select only the pixels which value is above it. The other pixels were deleted on each channel because are outside the range of interest.

**Figure 6:**
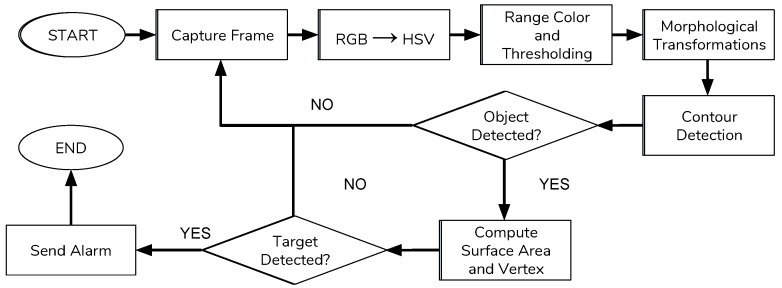
Flowchart for the detection process.

Morphological transformations were simple operations that are usually performed on binary images. These transformations needed two input parameters: the original image and a structuring element or kernel that specified the operation itself. Two basic operations were erosion and dilation and after these the open, close and gradient. These transformations were used to extract interesting features and to describe contours and surfaces. Erosion and dilation were filtering operations and were used to remove noise filling the holes that was present inside the image. The erosion was used to remove or thin objects inside a binary (white and black) image. The less important details were removed by computing the lowest number of pixels with the kernel overlap necessary to maintain the information consistent. In this way the smallest elements that were probably only noise were removed from the image while only the largest that are probably interesting remain. The dilation operator was used instead to enlarge or thicken objects inside a binary image. The intensity of the operation depends on the structure of the kernel. As opposed to erosion, this operation search for the maximum number of pixels inside the image with the kernel overlap. In this way, empty slots were filled and white spaces were thickened. After these operations, the contour detection was applied. This task was used to highlight the points inside an image in which brightness changes sharply. The output of this operation was a simpler image that contains fewer information than the original because the most part of unnecessary information is removed. Inside an image, the contour was simply a line that connected all the pixels with the same intensity features. After all these operations, the surface area, the length and the number of vertexes were calculated. If the area of the object is inside a predefined range of interest and the number of vertexes was four then an interesting object was detected inside the frame. If the detected object stayed inside the stream for several consecutive frames, then an alarm message was generated. A flowchart of the operations is shown in Figure 6.

## 4. Communication Issues

In this section, we discuss about the communication issues. In particular, several actors that interact in the environment are described. The main architecture core is represented by the edge nodes that are responsible for the elaboration on the data coming from cameras and sensors spread across the environments to monitor. Here, data are used for knowledge purposes. Taking into account information grabbed by those data, edge nodes decide to either command a drone to move in a specific area or to start a new activity such as patrolling or so on. The drawback of this organization is how the involved nodes communicate each other. In this work, we have chosen the MQTT protocol to carry messages between nodes. In the MQTT protocol we have three entities the broker, the subscriber and the publisher.

- The broker: it has the main goal to manage the messages coming from publishers and notify to the subscribers about the contents received. Several policies exist to manage the messages by the broker. In this work, the broker is placed in the edge layer and it can be reached by each node of the network. The broker we used is the MQTT Paho which is an open source broker available for free [38]. It has been installed in a server of the Telecommunication Laboratory in the University of Calabria, and it is available 24/7.
- Publishers and subscribers: In this work all the entities on different layers are both publisher and subscribers of some topic. To better depict the behavior of the whole system some of the messages and topics are herein described:
  – Status: status messages are sent by all devices involved in the environment. It contains the state of the device, the time-to-live, current operations. This message is sent periodically by devices. The destination of this message is the edge node that extracts data and stores it;
  – Data upload: data message is sent by the devices which are involved in sensor activities;
  – Commands to drone: messages that are sent to a specific drone are published on three different topics: remote control topic, surveillance topic and camera alert topic. On the remote control topic only messages to maneuver the drone from a remote user are sent. These messages are then encapsulated in a serial communication protocol and sent to the Arduino controller that manages the movements of the drone. The messages are described in Table 2. Each message is composed by a mandatory id and command and optional directions and options. The presence of the direction and options field depends on the type of command.
  – Commands to camera: the commands that can be received by a camera are useful to focus on some areas of interest where some anomaly is detected. These commands include some movement of the camera if the camera is not fixed, the resolution change of the video stream when more detailed frames are needed and also the chance to take a picture for high resolution analysis. These messages, as for the drone, are received on the specific topic camera control. The messages from each camera are then analyzed from the Raspberry controller which also send the command to the connected camera through the camera api interface. The commands that can be executed from a camera are shown in Table 3 and are similar to the drone commands;
  – Anomaly detected: when a camera or a drone detects something suspicious it notifies the hidden layer with a message published on the topic anomaly detection. When this message is received by the hidden layer it starts the coordination of other devices to focus the attention on the area of interest reducing in this way the time necessary to respond to a warning situation. These messages contain the device id that detected the anomaly and the position of the area of interest with a time-stamp label. The position is necessary for the hidden layer to coordinate in the right way the other devices. For example if an anomaly is detected in an area with two cameras that can change their angle then the position is necessary to send the right adjustment commands to them in order to focus correctly on the anomaly.

**Figure 7:**
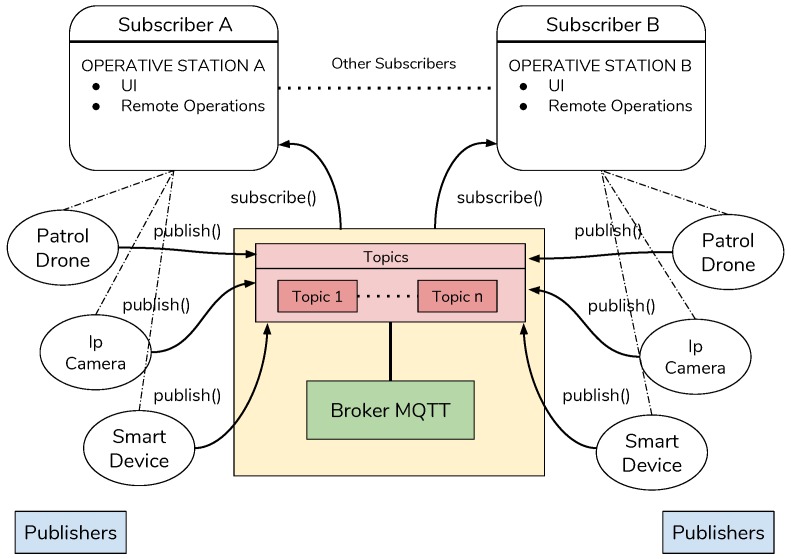
Machine-to-machine (M2M)-based communication architecture.

Taking into account the architecture layers depicted in Figure 7, it is possible to assign each message to a specific source layer. In particular, we defined three different layers with different assignments. Each layer is composed of different devices that have in charge some tasks.

## 5. Performance Evaluation

To validate the overall system we performed several tests. We built a test-bed for performing controlled test campaigns in our laboratory. The main goal of these campaigns was to evaluate how decentralized system performs in terms of response time and scalability. Moreover, we evaluated the performances of system comparing it with a cloud-edge system by moving in the higher layers some of the main functions such as computer vision tasks and management tasks. In particular, we moved the computer vision tasks as well as the data filtering, aggregation and analysis tasks on edge layer, whereas, we decided to move system management functions on the cloud layer.

### 5.1. Test Bed Description

**Figure 8:**
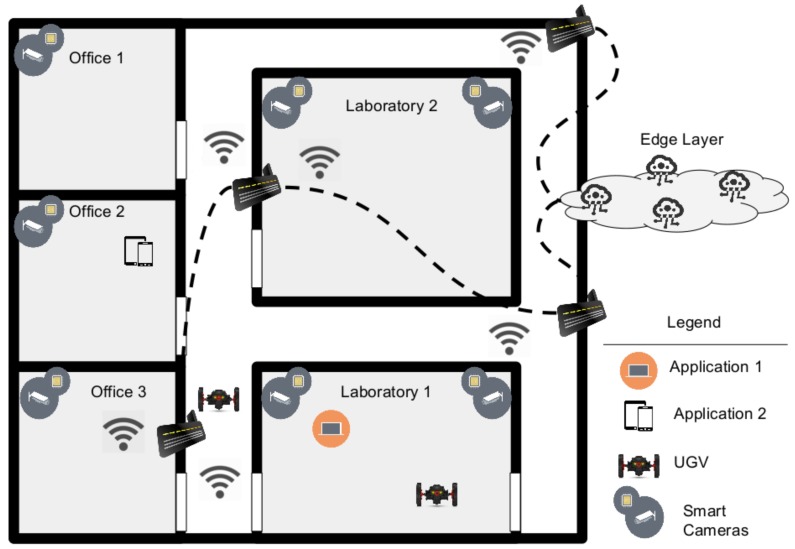
Test-bed configuration used for performing test campaigns in a controlled environment.

In this section, we described the test-bed we used for performing our performances evaluation campaigns. The test-bed was composed of smart IP cameras, each cameras has been equipped with a Raspberry Pi-3 board able to implement designed CV algorithms and protocol interfaces. Furthermore, we installed the Raspbian OS [46] and the OpenCV framework [47] for embedded devices on each Raspberry device. We used a mobile drone built for performing path-rolling in the indoor areas. Each area is delimited by our office rooms and laboratories. In each room we installed cameras able to communicate with edge layer and other devices. The Figure 8 depicts the test-bed we built up for performing campaigns. We controlled and monitored the system by using two applications: one on desktop PC and another one on mobile one.

#### Detailed Configuration

In this subsection, we supply some important details on the testbed configuration we used. The full environment we shown in Figure 8 is covered by WiFi connection thanks to several AP connected each other. Therefore, the device’s connections were guaranteed always. Cameras were connected in a wired manner as well as Raspberyy Pi in which CV modules lie. The edge layer, instead, was supplied by our server farm reachable by external connections. Therefore, the data stream had to pass through gateway. The edge layer was composed of three server machines one for DB, one for elaboration and the last one is used in case of traffic overload. All servers were linux-based and they provided low elaboration latency. The raspberry Pi3 device was equipped with Raspbian OS and with OpenCV 2.3 framework [47], moreover, it makde available python [48] and OpenJdk 8 [49] for calling framework API. In this case study we choose to call the framework by using python modules. Moreover, we implemented also MQTT calls primitive with a dedicated concurrent threads for sending and receiving messages from the broker. Regarding the storage capability, we used a local DB by using an Sqlite [50] instance. We implemented a driver wrapper in a dedicated thread, thus it will be possible to continue operation in a concurrent way avoiding to stop computational jobs by some instances that may require lock for a longer time. Regarding the Raspberry devices utilized to create the IoT camera device, we chose the Raspberry Pi for building up our prototype because of its feasibility and the possibility to use a linux based system for using the latest software technologies such as java, pyhton and common library for video analytics such as OpenCV. Anyway, it was possible to implement algorithms we used by building up dedicated embedded hardware for increasing the performances of the board. On the other hand, to realize the UGV, we used an Arduino board to create the control module. Moreover, we used a raspberry Pi3 for instantiating the communication module. As mentioned before the communication is made by using the presented customized protocol based on MQTT. The human interaction Layer was instantiated on a local PC installed in the room labelled Laboratory 1 in the Figure 8. For creating the front-end we use a web-based application connected with a cloud server instance that permits to use MQTT for connecting with the UGV and smart cameras. We use the node-red software [51] for creating high-layer interfaces on all of instances.

### 5.2. First Campaign: Detection Time

**Figure 9:**
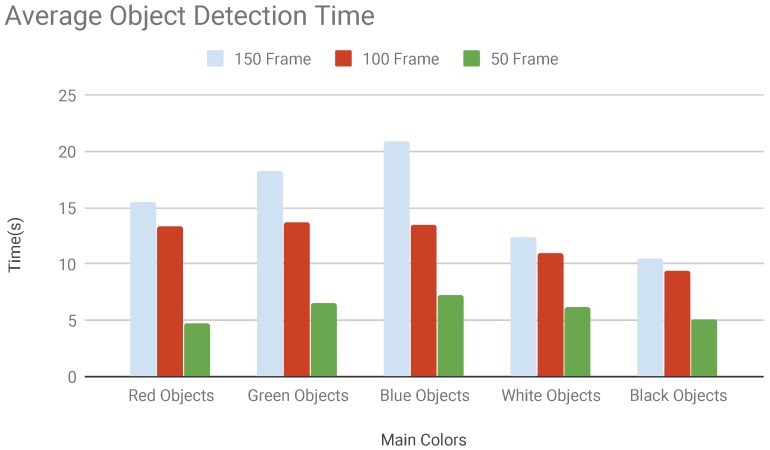
Average object detection time vs. number of frames.

The first campaign has been used to evaluate the ability of the system to recognize objects and to measure the number of frames necessary to detect the objects. Three tests have been performed where the objects were present inside 150 consecutive frames, 100 consecutive frames and 50 consecutive frames. Using 150 consecutive frames the time needed to detect the objects was very high for the three main colours while are lower for white and black objects. Diminishing the frame number also the detection time is lesser. When using a low number of consecutive frames the detection time of coloured and black and white objects are alike Figure 9. This happened because of the short duration of the stream which has a lower probability to present chromatic or brightness variations.

**Figure 10:**
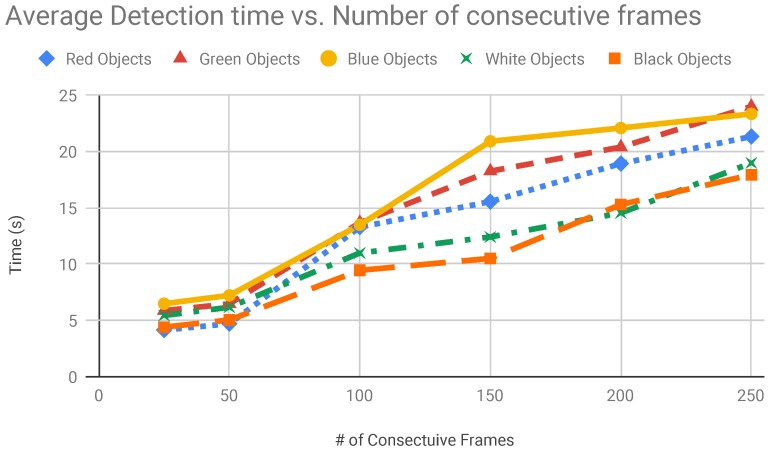
Average object detection time vs. number of frames.

The increment of the consecutive frames number affects the time for object detection. To find a good number of consecutive frames we tested different configuration for each colour. We started from 20 frames until we had 250 frames. The results of these tests are shown in Figure 10.

### 5.3. Second Campaign: Working Load

**Figure 11:**
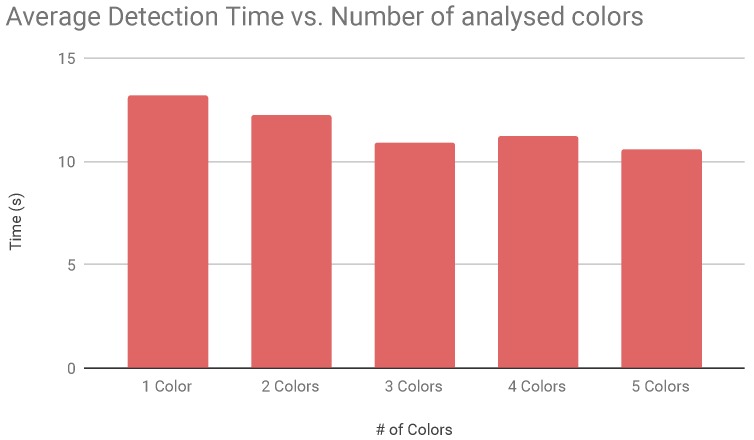
Average object detection time vs. number of colours.

In the second campaign we focused on the working load of the detection algorithm. We tested the time necessary for the detection of an object while searching for different colours inside the frame. For this test we used the red colour to be detected because it has a larger chromatic spectrum range. The detection time of objects is quite similar but we noticed a lower time when adding to the search algorithm other colours. This results are depicted in Figure 11.

### 5.4. Third Campaign: Shape Detection and False Positives

**Figure 12:**
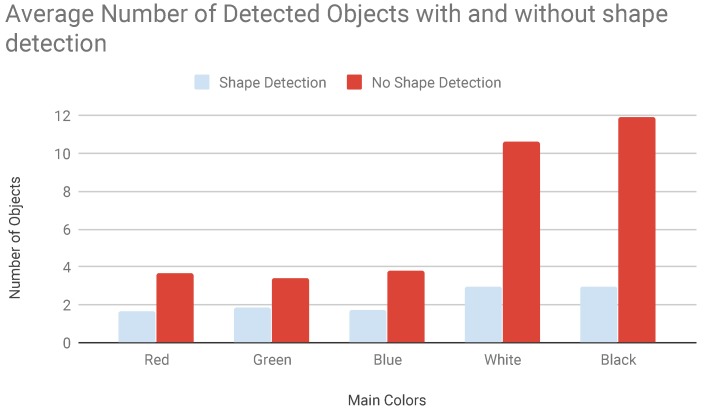
Average object detection with and without shape detection.

The third campaign focused on the filtering function of the shape detection and on how it enhances the quality of the recognition. The number of recognized objects inside consecutive frames differed because the shape detection features reduced false positives caused by brightness an colour changes inside the stream. The number of detected objects was halved when detecting colours while, one third in case of black and white objects. This happened because due to artificial lights where there was a great brightness discontinuity inside the video stream. The shape detection takes into account both number of vertexes and estimated surface area of the object. Our results are shown in Figure 12.

#### System Comparison

**Figure 13:**
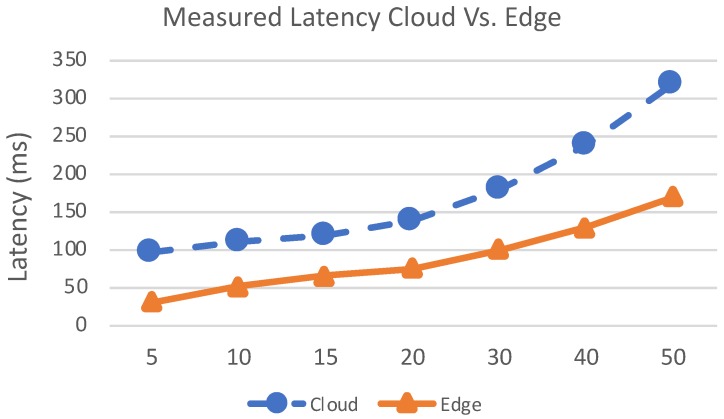
Measured latency on edge and cloud system configuration.

**Figure 14:**
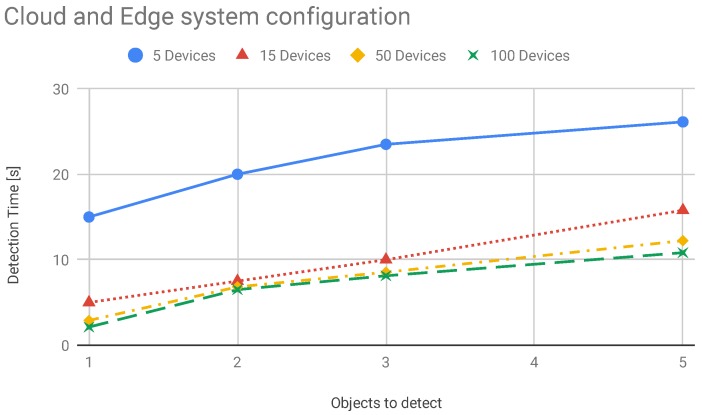
Average detection time vs. edge/cloud system configuration. Edge and cloud nodes are in charge of the tasks needed to perform data analysis coming from sensors devices such as IP cameras.

**Figure 15:**
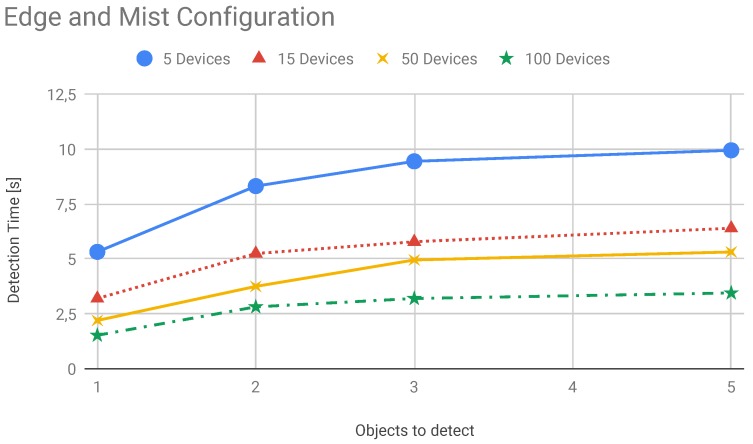
Average detection time vs. fully decentralized system configuration. Here, smart IP cameras are taking in charge of the detection process. Edge nodes receive post-elaborated data with information about detected objects.

**Figure 16:**
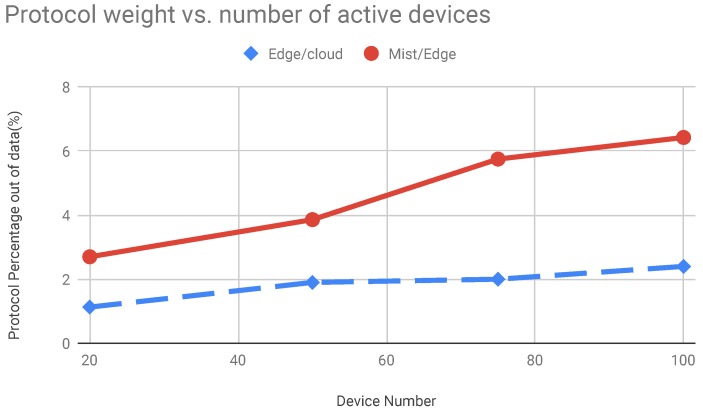
Protocol percentage out of data messages overall sent in the network by devices. We measured the total amount of bytes sent by devices active in the network and the protocol byte sent by devices.

In this section, we present the results about system scalability. We performed several tests to carry out these results. This scenario has the primary goal to demonstrate the goodness of the proposed system comparing it with a cloud based system. Even in this scenario, we verified how the system performs comparing it with a hybrid solution. Here, we move computational modules on edge nodes and distribute tasks between edge and cloud. We measured latency in edge and cloud system configuration for evaluating performances of the systems. In particular, we evaluate how the edge configuration can improve system performances when the number of the smart devices increase. The latency trend is shown in Figure 13. With the increasing of the number of devices the lambda value increases as well. This means that a higher number of services requests reach the edge and the cloud nodes. Therefore, the service rate decreases and the average measured latency increases. The latency is depicted in the Figure 13. In Figure 14 we reported the performance of the system composed of cloud and edge computing and management modules. In particular, we move the detection modules on edge nodes and the management modules on the cloud platform. In this way, before the system acts a strategy to solve found treats it has to wait the computation made by edge nodes. In this scenario, the edge node received the image captured by cameras and then perform detection algorithms. We have to take under consideration that these last algorithms are executed concurrently by the edge node. Therefore, we have to spend more time than a dedicated node to achieve the same results. Once an edge node detects a treat, represented by a recognized dangerous object, the information is passed to the cloud which will perform the decision-making algorithm. The cloud will be responsible for supporting action on the field by communicating the position of the alert source to the mobile drones by exploiting the M2M interfaces built on MQTT protocol. Instead, in Figure 15 we showed the behaviour of the system when smart IP cameras are used. In that case, the IP cameras perform CV analysis by their own and then report the analysis output to the edge nodes that have in charge the management duties. Now it is possible to note the detection time is lower than the edge/cloud system configuration already depicted in the Figure 14. Moreover, when increasing the number of objects to detect it is possible to note that the slope of the detection trend is much more smoothly than the trends shown in Figure 14. In the Figure 16 we measured the performances of the system in both configuration we already mentioned before. In particular, we evaluated an index achieved as the ratio of the total amount of protocol bytes sent to the hidden layer by active devices out of the total bytes sent. The total bytes are obtained by adding the protocol bytes and data bytes. In the Figure 16 it is possible to observe how the edge/cloud configuration outperforms the mist/edge configuration in terms of control messages sent on the network. We depicted the ratio between the overall control protocol messages and the total messages sent. Thus we achieve the percentage as shown in Equation (8).

(8) $$pp=\frac{T_{ptr}}{T_{ptr}+T_{data}}\times 100$$

Here, the pp term means protocol percentage, this term is reported on the ordinate axis of the Figure 16. Moreover, $T_{ptr}$ term reports the total control protocol message sent by smart devices and computation nodes on the network, at the same way we defined the $T_{data}$ term that reports the total number of data messages sent on the network by the smart devices. It is possible to note that the the mist/edge configuration uses more bytes for protocol purpose out of the data bytes than the edge/cloud configuration. This happen because of the camera streaming that is used by the edge/cloud configuration. Indeed, in this configuration devices send data to the edge layer for further elaboration.

## 6. Conclusions

In this work, we presented a decentralized solution based on edge and mist computation in a specific application area. We demonstrated that a distributed computational architecture perform better than centralized architecture in terms of resource allocation, system response time and scalability. In this work we also demonstrated that the edge computing can be supported by mist computing for increasing the overall system performances. In particular, we provided some test campaigns that proved the goodness of the proposal in terms of scalability and computational time needed for achieve results. The proposal system has been compared with another approach based on edge and cloud computing. We achieved good performances also evaluating networking parameters such as protocol overhead and network resources. In this work, we proposed analysis on an evaluation of the system latency in scenarios that offer only cloud services versus scenarios with decentralized system architecture. The report showed that the system with a decentralized configuration performs better with a gain of 30% in the best condition and an increase of 5% in the worst scenarios in which user requests overload all edge node; this happens because the edge nodes have fewer resources than the cloud solutions, and they may measure congestion quicker than the cloud. Regarding future work, we are thinking to improve this proposal by focusing on the development of the embedded hardware. We are planning to use an open hardware solution based on ARM microcontroller compliant with a Linux arm based operating system (OS). We are improving mist computation modules by performing an in-depth analysis of the data we can collect. We are moving some data aggregation module and artificial neural network (ANN) modules for making the device cognitive.

## Figures and Tables

**Table 1:** Symbols in the mathematical formulation.

Symbol	Description
λ	arrival rate
μ	service rate
*c*	number of available servers
*V*	computing latency per task
$d_{comp}$	computation delay
*f*	instructions per seconds
*K*	number of instructions required per task
$d_{node}$	delay in a computation node
*W*	queuing delay
ρ	utilization factor
$P_{Q}$	queue probability
$L_{cloud}$	cloud based latency
$L_{edge}$	edge based latency
α	proportional constant for uplink and downlink from the smart device to AP
β	proportional constant for uplink and downlink from AP to the cloud
$d_{ue,AP_{i}}$	distance from *i*-th AP to smart device
$d_{AP_{i},cloud}$	distance between *i*-th AP to the cloud
*m*	number of available AP
$d_{s}$	delay for overload on the edge node

**Table 2:** Movement commands received from the Arduino controller.

id	Command	Directions	Options
Device	Move Stop Speed Turn Rotate Surveillance AlarmDetected RemoteControl	forward backward left right up down	throttle or digital value for speed

**Table 3:** Commands that can be received by a camera controller.

id	Command	Directions	Options
Device	Rotate TakePicture ChangeResolution Zoom	left right up down in out	decimal valute that can represent the degrees for the rotation, the percentage of zoom or the resolution

## Abbreviations

The following abbreviations are used in this manuscript:

IoT	internet of things
FSM	finite state machine
CV	computer vision
MQTT	message queuing telemetry transport
H2M	human-to-machine
M2M	machine-to-machine
UGV	unmanned ground vehicle
OS	operating system
ANN	artificial neural network
AP	access point
LTE	long term evolution
LTE-A	long term evolution-advanced
MTC	machine-type communication
HTC	human-type communication
LDWS	lane departure warning system
OFDMA	orthogonal frequency-division multiple access
H2H	human-to-human
QoS	quality of service
CPS	cyber-physical system
ANN	artificial neural network
IoT	internet of things
TTL	transistor–transistor logic
SDN	software defined networking
FCFS	first come first served
AP	access point
